# Low Dose of IL‐2 Application for Graft‐Versus‐Host Disease Prophylaxis Following Haploidentical Stem Cell Transplantation

**DOI:** 10.1002/mco2.70587

**Published:** 2026-01-15

**Authors:** Zheng‐Li Xu, Meng Lv, Xing‐Xing Yu, Yi‐Yang Ding, Ting‐Ting Han, Hai‐Xia Fu, Yuan‐Yuan Zhang, Xiao‐Dong Mo, Yu‐Qian Sun, Lan‐Ping Xu, Xiao‐Hui Zhang, Yu Wang, Xiao‐Jun Huang, Xiang‐Yu Zhao

**Affiliations:** ^1^ Peking University People's Hospital Peking University Institute of Hematology National Clinical Research Center for Hematologic Disease Beijing Key Laboratory of Cell and Gene Therapy For Hematologic Malignancies Beijing China; ^2^ Collaborative Innovation Center of Hematology Beijing University Beijing China

**Keywords:** graft‐versus‐host disease, haploidentical transplantation, interleukin‐2, natural killer cells, T‐regulatory cells

## Abstract

Graft‐versus‐host disease (GVHD) are still key obstacles of haploidentical transplantation. Interleukin‐2 (IL‐2) could promote natural killer (NK) cells and T‐regulatory cells (Tregs) cells expansion in vitro and in vivo. We explored whether low‐dose IL‐2 administration at an early stage could promote NK cells and Tregs reconstitution and reduce GVHD after haplo‐HSCT. This cohort trial included 10 recipients of accepting IL‐2 treatment and case‐pairing 30 recipients without IL‐2 treatment post haplo‐HSCT. In contrast to the control group, the 5‐year incidence of chronic GVHD (cGVHD) was lower (*p* = 0.018), and GVHD progression‐free survival (GPFS) was better (*p* = 0.025) in the IL‐2 group. Blood NK‐cells, Treg cells, conventional T cells (Tcon) cells, and the expression of CD62L+ on Tregs and Tcon cells reconstitution were increased post‐IL‐2 treatment. NKG2A expression on NK cells increased significantly post‐IL‐2 treatment. Meanwhile, IL‐2 administration shortly increased the plasma levels of IFN‐Ƴ, TNF‐a, IL‐10, and IL‐2 in subjects post haplo‐HSCT. Relative to the control group, low‐dose IL‐2 increased NK cell counts and the expression of CD122, DNAM‐1, and NKG2D on NK cells post transplantation. Administration of low‐dose IL‐2 after haplo‐HSCT correlated with reduced cGVHD, which should be explored further with randomized trial.

## Graphical abstract

Early low‐dose IL‐2 administration after haploidentical HSCT promotes the reconstitution of NK cells and regulatory T cells (Tregs), enhances their activation (CD122^+^, DNAM‐1^+^, NKG2D^+^, CD62L^+^), and increases plasma cytokines (IFN‐γ, TNF‐α, IL‐10, IL‐2). These immune modulations are associated with reduced chronic GVHD incidence and improved GVHD progression‐free survival.

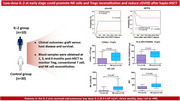

## 1 Introduction

Graft‐versus‐host disease (GVHD) persists as a significant challenge in the setting of allogeneic hematopoietic stem cell transplantation (allo‐HSCT) [1]. Natural killer (NK) cells and T‐regulatory cells (Tregs) could regulate alloreactive T‐cell function to decrease the incidence of GVHD. Being the first lymphocyte subset to recover after allo‐HSCT, NK cells are crucial mediators of early immune responses [2]. Lower Day 14 NK cell numbers predicted a higher incidence of the incidence of Grades 2–4 acute GVHD (aGVHD) [3]. Mice model indicated that NK cells adoptive infusion before transplantation could prevent the occurrence of aGVHD [4, 5]. Meanwhile, CD4+CD25+ regulatory T cells (Tregs) can also inhibit GVHD while maintaining graft‐versus‐tumor activity in mice model [6].

Ultra‐low‐dose interleukin‐2 (IL‐2) enhances immunomodulating function of Treg cells and NK Cells in healthy volunteers [7]. IL‐2 critically supports Treg cell development and maintenance while mediating activation‐induced cell death, thereby fostering tolerance and suppressing inappropriate immune responses [8]. IL‐2 could also promote cytolytic activity in CD8+ T cells and NK cells [9, 10]. Ultra‐low‐dose IL‐2 administration has good tolerability and drives Treg population expansion in vivo [11] and may be linked to a decreased incidence of infections and GVHD after HLA‐matched related or unrelated allo‐HSCT [12].

Our previous controlled, open‐label randomized trial has indicated that a low dose of IL‐2 administration at Day 60 reduced the incidence of chronic GVHD (cGVHD) and translated into improved GVHD progression‐free survival (GPFS) post allo‐HSCT [13]. Recipients receiving low‐dose IL‐2 demonstrated elevations in circulating Treg and NK cell populations, together with augmented NK cytotoxic function [14]. These results indicated that early post‐transplantation low‐dose IL‐2 was associated with decreased cGVHD, yet had no effect on relapse incidence.

On the basis of this evidence, we undertook a prospective cohort trial to investigate whether early low‐dose IL‐2 after haploidentical HSCT is both safe and effective in reducing acute or cGVHD rates.

## 2 Results

### 2.1 Study Population

One hundred twenty‐four consecutive patients who underwent haplo‐HSCT between April 2016 and April 2017 were screened. Among these, 10 patients approved participation in IL‐2 treatment, and another 30 persons were recognized as the control group accepting the conventional haplo‐HSCT protocol with equivalent patient and donor characteristics matched as diagnosis, sex, age, risk category, and donor–recipient relationship (Table 1).

**TABLE 1:** Baseline characteristics between IL‐2 group and control group.

Characteristics	IL‐2 group (*n* = 10)	Control group (*n* = 30)	*p*‐value
**Patient age at HSCT, years, median (range)**	31.5 (17–46)	30 (15–50)	0.866
**Disease types, *n* (%)**			0.652
AML	3 (30.0%)	14 (46.7%)	
MDS	3 (30.0%)	7 (23.3%)	
B‐ALL	4 (40.0%)	9 (30.0%)	
**Patient‐donor gender, *n* (%)**			0.357
Male–male	4 (40.0%)	15 (50.0%)	
Male–female	3 (30.0%)	10 (33.3%)	
Female–male	2 (20.0%)	5 (16.7%)	
Female–female	1 (10.0%)	0	
**Source of donor, *n* (%)**			0.722
Father	4 (40.0%)	14 (46.7%)	
Sibling	4 (40.0%)	8 (26.7%)	
Child	2 (20.0%)	8 (26.7%)	
**HLA‐A, ‐B, ‐DR, mismatches, *n* (%)**			0.408
0–2	2 (20.0%)	3 (10.0%)	
3	8 (80.0%)	27 (90.0%)	
**Patient CMV serology pre‐HSCT**			0.402
Positive	10 (100%)	28 (93.3%)	
Negative	0	2 (6.7%)	

The median duration of the subjects accepting IL‐2 treatment was 14 days (range 1–77 days). The median number of IL‐2 administrations was seven times (0.4 × 10^6^ U/m^2^/time) with a range of 1–33 times. Only three patients accepted the full treatment of 77 days. The remaining seven patients had to stop IL‐2 treatment because of fever (*n* = 6), and intolerant subcutaneous nodules (*n* = 1), all graded as CTCAE Grade 1.

### 2.2 GVHD

**FIGURE 1:**
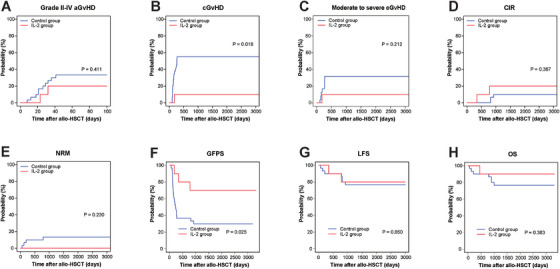
The clinical outcomes between the IL‐2 and control arms.

Five, two, and zero patients in the IL‐2 cohort developed Grades 1–4, Grades 2–4, and Grades 3 and 4 aGVHD, compared with 17, 10, and five patients in the control cohort, respectively. Median time to developing aGVHD was 32 days in the IL‐2 group and 23.5 days in the control group. The onset time of aGVHD had a trend to be postponed in the IL‐2 group (*p* = 0.082). All the patients developed aGVHD post stopping IL‐2 treatment. The cumulative incidences of Grades 2–4 (20.0% [0%–46.2%] vs. 33.3% [16.1%–50.6%], *p* = 0.411, Figure 1A) and Grades 3 and 4 (0% vs. 16.7% [3.1%–30.2%], *p* = 0.174) aGVHD in the IL‐2 group were comparable to control group.

cGVHD developed in one patient from the IL‐2 group versus 16 subjects from the control cohort. The cumulative incidence of cGVHD (all stages) was decreased in the IL‐2 cohort than in the control cohort (10% [0%–29.6%] vs. 55.2% [36.5%–73.8%], *p* = 0.018, Figure 1B). Moderate‐to‐severe cGVHD appeared to be lower in the IL‐2 group, but the difference was not statistically significant (10% [0%–29.6%] vs. 31.7% [11.5%–52.1%], *p* = 0.212, Figure 1C). The multivariate analysis revealed that early post‐transplantation IL‐2 treatment obviously decreased the incidence of cGVHD, compared with the control arm (HR = 0.121; 95% CI, 0.016 to 0.913; *p* = 0.041).

#### 2.2.1 Survival

The 5‐year cumulative incidence of relapse (CIR) in the IL‐2 cohort was 20.0% (0%–46.2%) versus 10% (0%–21%; *p* = 0.367, Figure 1D) in the control arm. No NRM deaths occurred in the IL‐2 group, compared with four in the control group. Two subjects died of severe aGVHD, and two other subjects died of Intracranial infection. The cumulative incidence of NRM rates had a trend to be decreased among patients with IL‐2 administration (0% vs. 13.3% [0.1%–25.7%]; *p* = 0.230, Figure 1E).

There were no significant differences in CMV viremia (100% vs. 90% ± 5.5%, *p* = 0.963) or EBV viremia (30% ± 14.5% vs. 40% ± 8.9%, *p* = 0.564) between IL‐2 and control cohorts.

Five‐year GPFS outcomes were significantly better in the IL‐2 group than in the control cohort (70.0% [46.7%–100%] vs. 30.0% [17.4%‐51.8%], *p* = 0.025, Figure 1F). The 5‐year LFS rates was 80.0% (58.7%–100%) in the IL‐2 cohort vs. 76.7% (62.9%–93.4%) in the control cohort (*p* = 0.850, Figure 1G), and the 5‐year OS were 90% (73.2%–100%) vs. 76.7% (62.9%–93.4%) in the IL‐2 and control groups (*p* = 0.383, Figure 1H).

### 2.3 Impact of Low‐Dose IL‐2 on Tregs Reconstitution

**FIGURE 2:**
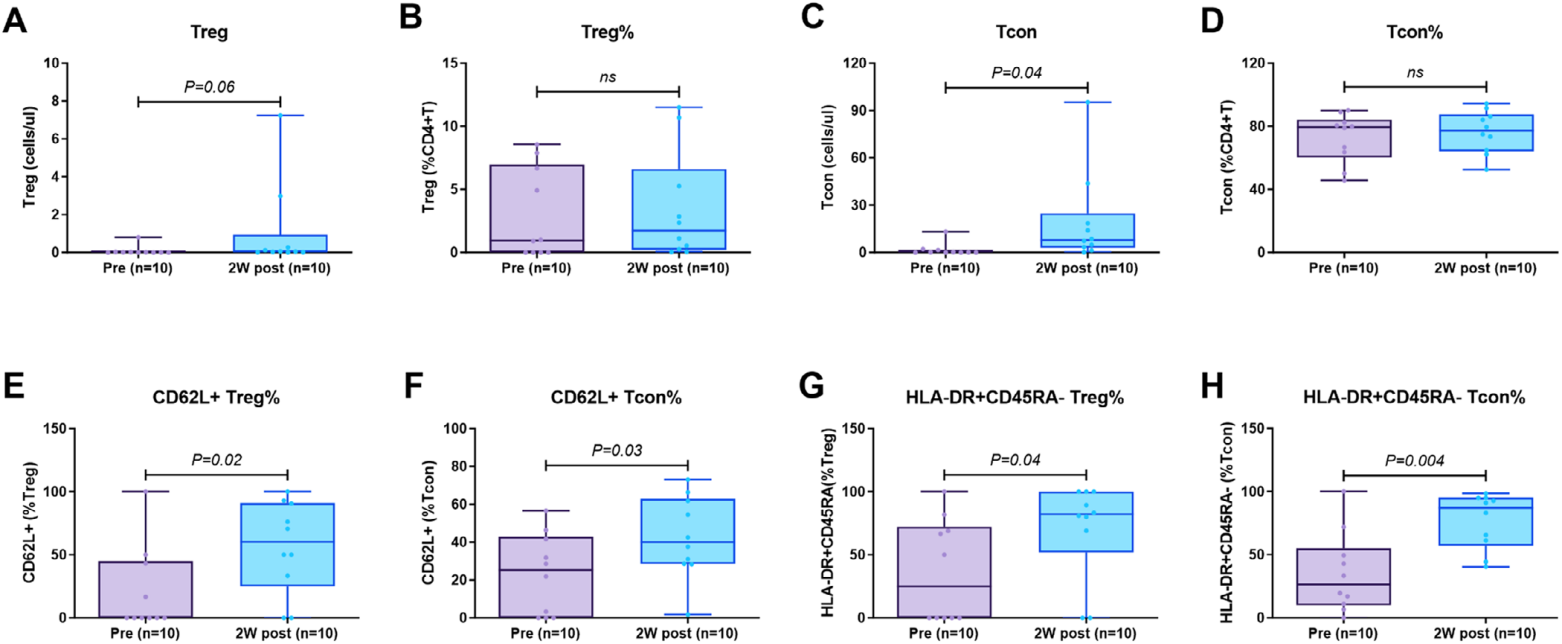
Reconstitution of peripheral blood Treg cells and NK cells at two time points post‐transplantation before and post‐IL‐2 treatment.

**FIGURE 3:**
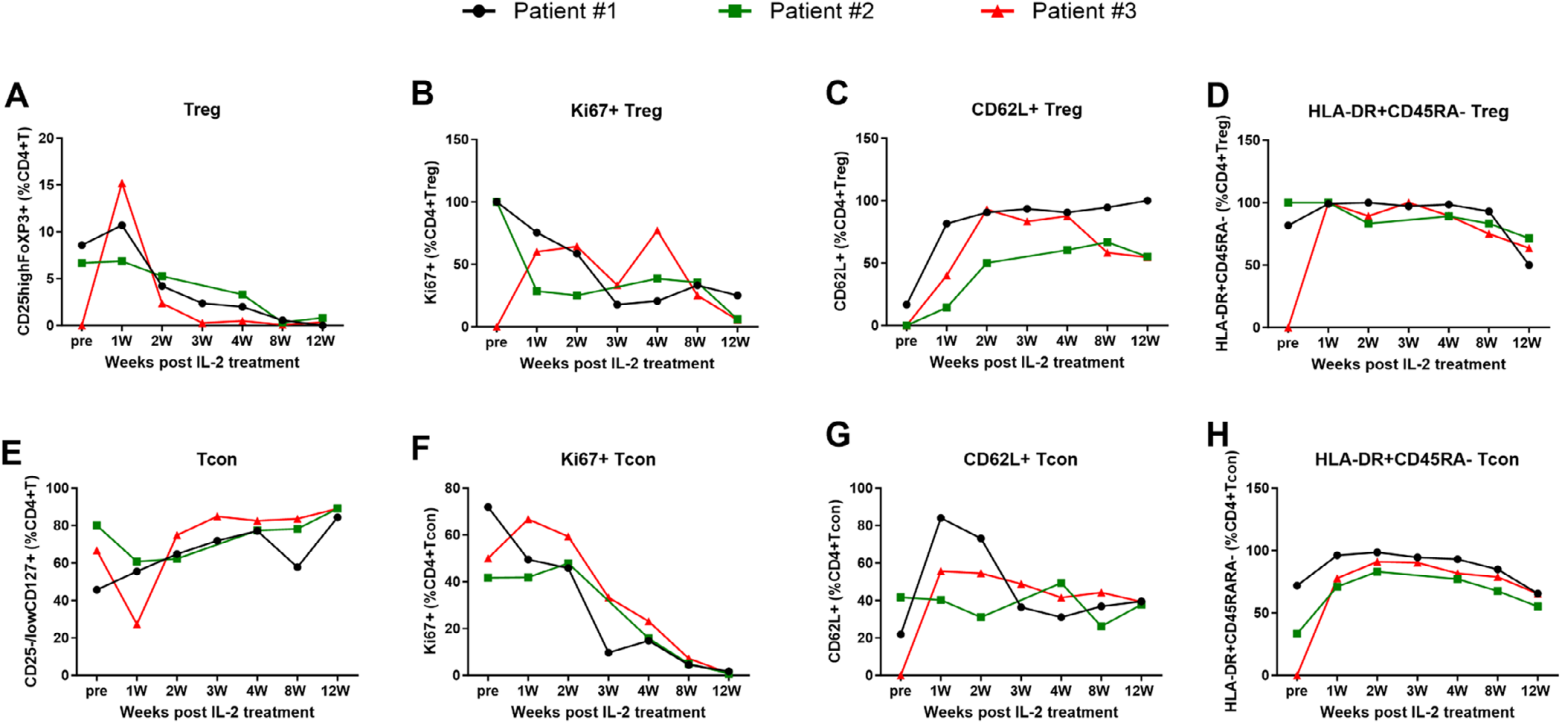
Continuous change of CD4^+^CD25^high^CD127^−/low^FoxP3^+^ Treg cell low dose of IL‐2 treatment.

The absolute numbers of CD4^+^CD25^high^CD127^−/low^FoxP3^+^ Tregs (T_reg_) cells and CD4^+^CD25^low^CD127^high^ conventional T cells (Tcon) were increased post 2 weeks IL‐2 treatment, mainly with the increase of the HLA‐DR+CD45RA‐ subset among Treg or Tcon cells. The expression of CD62L on Treg or Tcon cells was significantly increased post 2 weeks of IL‐2 treatment (Figure 2). However, the expression of CCR7 on Treg or Tcon cells was very low and had no significant change post‐IL‐2 treatment. No significant change was found on the expression of Ki67, Helio, and CD95 expression on Treg or Tcon cells post‐IL‐2 treatment (data not shown). The continuous change of Treg and Tcon as well as NK cells among the three patients who completed the entire course of IL‐2 treatment are shown in Figure 3.

### 2.4 Impact of Low‐Dose IL‐2 on NK Cell Reconstitution

**FIGURE 4:**
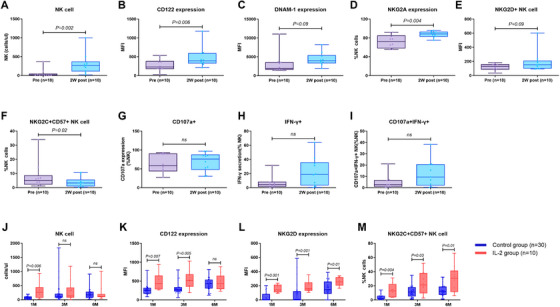
NK cell reconstitution is expanded by a low dose of IL‐2.

The absolute numbers of NK cells were increased post 2 weeks of IL‐2 treatment. The proportions of CD56^bright^ and CD56^dim^ NK cells were comparable before and post‐IL‐2 treatment. The expression of IL‐2Rβ (CD122), inhibitory receptor NKG2A, adherence molecules DNMA‐1 and activating receptor NKG2D on NK cells was increased post‐IL‐2 treatment (Figure 4A–D). The expression of IL‐2Rα (CD25) and inhibitory receptor KIRs (KIR2DL1, KIR2Dl2/L3, KIR3DL1) as well as the activation receptors NKP30 or NKP46 on NK cells was comparable before and post‐IL‐2 treatment. CD107a and IFN‐γ expression by NK cells stimulated with K562 cells was comparable pre‐ and post‐IL‐2 treatment. However, the NKG2C+CD57+ NK cells were decreased at 2 weeks post‐IL‐2 treatment, then increased rapidly 1 month after transplantation. Therefore, compared to the control arm, the percentage of NKG2C^+^CD57^+^ NK subsets among NK cells as well as the expression of CD122, DNAM‐1 expression on NK cells was markedly elevated in the IL‐2 arm, compared to the control arm. However, CD107a and IFN‐γ expression by NK cells against K562 cells were similar between the IL‐2 arm and the control arm (Figure 4).

### 2.5 Impact of Low‐Dose IL‐2 on Plasma Cytokines

**FIGURE 5:**
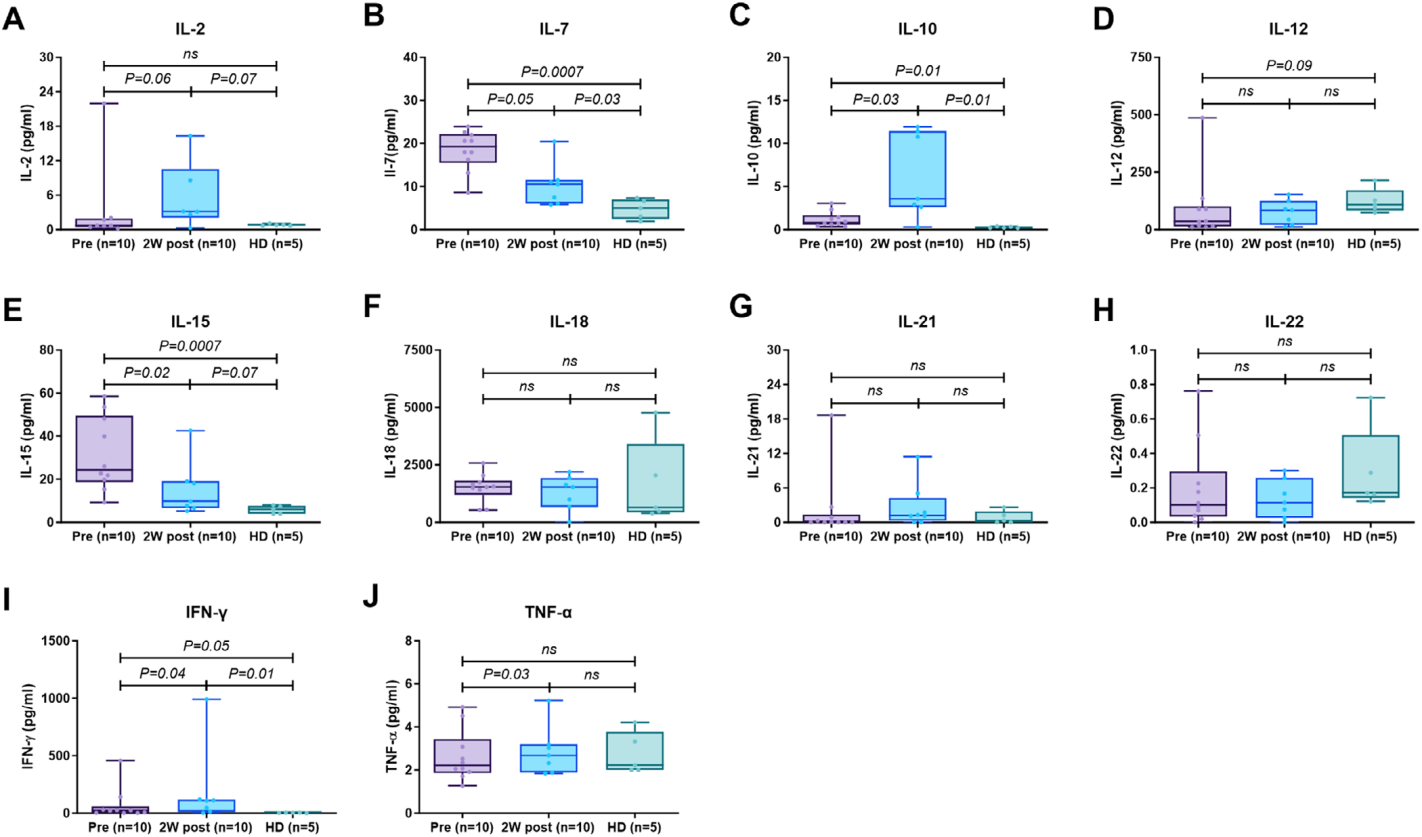
Plasma cytokines kinetics before and post‐IL‐2 treatment.

**FIGURE 6:**
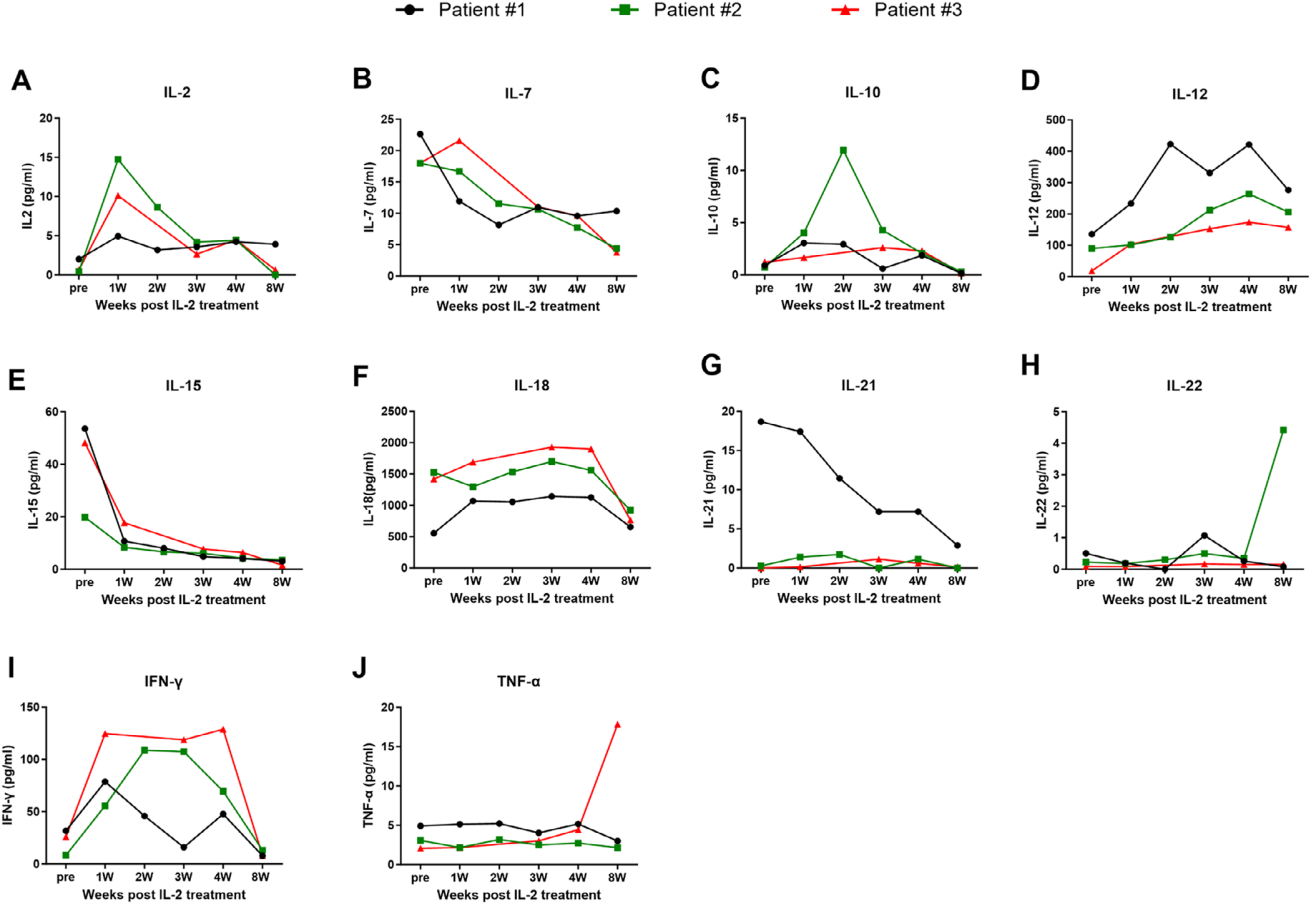
Continuous change of plasma cytokines kinetics before and post‐IL‐2 treatment.

Considering seven among the 10 patients could not complete the IL‐2 treatment because of fever, we screened the plasma cytokines before and post 2 weeks of IL‐2 treatment. As shown in Figure 5, compared to the healthy donors (HDs), the plasma cytokines levels including IFN‐γ, IL‐15, IL‐7, IL‐10, IL‐2, and IL‐12 were higher among patients before or post‐IL‐2 treatment. The levels of TNF‐α, IL‐22, IL‐21, and IL‐18 were comparable between patients post transplantation and healthy donors. Comparison between patients before and post‐IL‐2 treatment, IL‐2 treatment significantly increased the plasma levels of IFN‐γ, IL‐2, TNF‐α, and IL‐10 but not IL‐15, IL‐7, IL‐22, IL‐21, and IL‐18. The continuous change of plasma levels of the above cytokines among the three patients who completed the entire course of IL‐2 treatment is shown in Figure 6.

## 3 Discussion

Haploidentical transplantation has made great advances in the recent decade, with optimized GVHD prophylaxis [15]. This study investigated whether early post‐transplant prophylactic low‐dose IL‐2 reduces acute and cGVHD incidence by expanding NK and Treg cell populations. Our findings demonstrated that early IL‐2 administration did not decrease the incidence of aGVHD; rather, it may only postpone its onset. In fact, we observed a lower rate of cGVHD in subjects receiving IL‐2 at an early stage after allo‐HSCT. This represents the first description of this finding to our knowledge.

We previously established in a randomized controlled study that administrating IL‐2 during the intermediate post‐transplant period decreases cGVHD [13]. Notably, the present study demonstrates IL‐2 administration at early stage also reduced cGVHD incidence. Consistent with published data [12, 16], IL‐2 treatment profoundly expanded Treg cells. Similar with the prior report [17], Treg numbers inversely correlated with cGVHD incidence, suggesting IL‐2 may protect against cGVHD through expansion of these immunoregulatory cells. Koreth et al. demonstrated that low‐dose IL‐2 effectively improves cGVHD symptoms [16, 18]. Subsequently, they proved infusion of polyclonal healthy donor Tregs followed by expansion with IL‐2 is safe among patients with steroid‐refractory cGVHD [19]. The above data have suggested both therapeutic and prophylactic roles in cGVHD post‐IL‐2 administration. Previous studies investigating Treg cell roles in cGVHD primarily used samples obtained after diagnosis. We prospectively evaluated allo‐HSCT patients receiving unmanipulated grafts to characterize the natural history of cGVHD with versus without IL‐2 administration, demonstrating the protective roles of Tregs and IL‐2 therapy with direct evidence.

Prior research has shown that during the first 30 days following transplantation, the majority of NK cells exhibit immunoregulatory rather than cytotoxic properties, characterized by the CD56^bright^ CD16 phenotype [15, 20]. In our cohort, IL‐2 administration significantly expanded CD56^bright^ NK cells. Cytotoxicity assays in vitro revealed that the expanded NK cells, independent of CD56^dim^ versus CD56^bright^ phenotype, could lyse both resting and activated Tcon. This implicates early CD56^bright^ NK cell expansion as a potential protective mechanism against cGVHD development. Prior work established that CD56^bright^ NK cells suppress T‐cell survival in a contact‐dependent manner [21]. Consistently, high NK cell numbers at 3 months after allo‐HSCT have been correlated with lower cGVHD rates [22]. Functional analyses revealed that expanded CD56^bright^ NK cells preserved IFN‐γ production, a key mediator of antimicrobial immunity that drives dendritic cell maturation and Th1 responses [23, 24]. Consistently, our prior work demonstrated that rapid CD56^bright^ NK cell reconstitution was associated with decreased transplantation‐related mortality [25]. Accordingly, IL‐2‐driven expansion of CD56^bright^ NK cells may confer dual benefits, including mitigating moderate‐to‐severe cGVHD and bolstering antimicrobial defense. While OS and LFS did not differ between groups, GPFS was markedly improved in the IL‐2‐treated cohort.

Numerous publications have reported an inverse link between cGVHD and relapse risk in acute leukemia [26, 27]. In this cohort, T‐ALL was excluded from the criteria due to concerns that IL‐2 might promote the proliferation of acute T lymphoblastic leukemia cells. AML and B‐ALL were both included to broaden applicability. Although NK cytotoxicity against K562 targets increased following HSCT, IL‐2 administration in vivo had no effect on leukemia prevention. These findings therefore prompt reconsideration of whether NK cells serve as the primary mediators of allogeneic anti‐leukemia activity. Hallett et al. recently reported that Treg depletion prior to IL‐2 administration conferred superior antitumor efficacy in long‐term tumor outgrowth models relative to either monotherapy [28]. Bachanova et al. demonstrated in an adoptive NK cell infusion trial that host Treg depletion with IL‐2 diphtheria toxin fusion protein (IL‐2DT) improved the efficacy of haploidentical NK cell therapy for refractory AML [29]. The elevated Tregs within the tumor microenvironment may suppress the GVL effects of NK cells and other cytotoxic T cells following transplantation [30]. In this study, no effect of IL‐2 on increasing leukemia relapse was observed.

This study has several limitations to date. This was an exploratory cohort study, and no formal power calculation was performed. As such, the study may be underpowered to detect differences in some secondary endpoints. This study was also limited by its open‐label, non‐randomized design, the absence of a placebo control, and potential selection bias. Moreover, the limited sample size limited the generalizability of these findings. Last but not the least, B‐cell reconstitution was not evaluated in this study but will be addressed in future work.

In summary, low‐dose IL‐2 administered at the early stage post‐transplantation was connected with a lower frequency of cGVHD and a better GPFS. Thus, prophylactic administration of IL‐2 should be considered among patients at high risk of developing cGVHD.

## 4 Methods

### 4.1 Eligibility Criteria

We conducted an open‐label, prospective cohort study at Peking University Institute of Hematology from April 2015 to April 2017. Patients aged 15–65 years with acute leukemia in complete remission (CR) who underwent myeloablative unmanipulated allo‐HSCT were eligible. Additional inclusion criteria included: (1) diagnosis with non‐T‐ALL; (2) underwent haplo‐HSCT; (3) neutrophil engrafted between Days 7 and 20 post transplantation, and (4) no severe infection or active aGVHD. The exclusion criteria included T‐ALL diagnosis, active infection, uncontrolled organ dysfunction, or prior IL‐2 therapy. This trial was registered on ClinicalTrials.gov (NCT00539695).

### 4.2 Study Design

Patients were enrolled prospectively into the IL‐2 group if they consented, controls were selected from a contemporaneous registry using matched pairing based on diagnosis, age, and donor type. No formal sample size calculation was performed because this was an exploratory Phase II study. Low‐dose IL‐2 was administered subcutaneously to patients in the IL‐2 arm (0.4 × 10^6^ U/m^2^, thrice weekly, Days +14 to +90) when neutrophil engrafted post allo‐HSCT. IL‐2 was discontinued in cases of persistent fever > 38.5°C, injection‐site intolerance, or patient request. The transplantation procedure for the control arm was identical, except IL‐2 was omitted. This study was approved by the Peking University Institutional Review Board; all patients gave written informed consent per the Declaration of Helsinki before enrollment. Ultimately, 30 and 10 patients were included in the control and IL‐2 arms, respectively.

### 4.3 Transplants

The conditioning for haploidentical transplantation consisted of: intravenous (IV) cytarabine 4 g/m^2^/day (Days −10 and −9), IV busulfan 3.2 mg/kg/day (Days −8 to −6), IV cyclophosphamide 1.8 g/m^2^/day (Days −5 and −4), oral Me‐CCNU 250 mg/m^2^ (day ‐3), and IV ATG 2.5 mg/kg/day (days ‐5 to ‐2; Sang Stat, Lyon, France) [31]. All patients received a combined graft of G‐CSF‐mobilized bone marrow (GBM) and peripheral blood stem cells (PBSC), harvested and infused per our previous protocols [32].

### 4.4 GVHD Prophylaxis and Treatment

GVHD prophylaxis consisted of cyclosporine A (CsA), mycophenolate mofetil (MMF), and short‐term methotrexate (MTX) for all recipients. CsA was administered IV at 2.5 mg/kg/day starting Day −3, with dose adjusted to maintain trough levels of 150–250 ng/mL, tapered, and discontinued 4–6 months post HSCT. MTX dosing was 15 mg/m^2^ IV on Day +1 and 10 mg/m^2^ on Days +3, +5, and +11 [33]. Oral MMF was administered (0.5 g twice daily) from Day −3 until neutrophil engraftment. aGVHD (aGVHD) was treated with first‐line methylprednisolone (1–2 mg/kg/day) and second‐line anti‐CD25 monoclonal antibodies [34, 35].

### 4.5 The Monitoring and Prevention of Cytomegalovirus and Epstein–Barr Virus

CMV and EBV viral loads were monitored, and infections were managed as previously described [36].

### 4.6 Definitions and Evaluation

Definitions for engraftment, viral infection, NRM, relapse, and survival outcomes followed established criteria [33]. Acute and cGVHD were evaluated per international consensus, using MAGIC criteria for aGVHD and NIH 2014 criteria for cGVHD [37, 38]. The occurrence of relapse was determined based on international criteria [33].

### 4.7 End Points

The primary endpoint was the cumulative incidence of safety and cGVHD. The secondary endpoints were aGVHD, CMV, EBV, NRM, OS, DFS, and GPFS.

### 4.8 Blood Sample Preparation and Immune Reconstitution Monitoring

A total of 40 subjects (IL‐2 cohort, *n* = 10; control cohort, *n* = 30) were monitored prospectively for Treg, conventional T cell, and NK cell reconstitution. Blood samples were obtained at 1, 3, and 6 months following HSCT, with the IL‐2 cohort receiving additional pre‐ and post‐treatment sampling to directly characterize treatment effects. Flow cytometry analysis was performed on all samples to identify CD3+ T cells, CD56+ NK cells, CD4^+^CD25^high^CD127^low/−^ Tregs, CD4^+^CD25^−/low^CD127^+^ Tcon, and CD4^+^CD25^+^Foxp3^+^ Tregs as well as the expression of CD31, HLA‐DR, CD45RA, BCl‐2, and Ki67 on Treg cells. Functional analysis of the NK cells against K562 cell were also monitored.

### 4.9 Statistical Analyses

Intergroup comparisons used the chi‐square test for categorical variables and Mann–Whitney U test for continuous variables. Using a competing‐risk model, we generated cumulative incidence curves to calculate NRM probabilities (relapse as competing event) and to assess GVHD, engraftment, EBV or CMV, and relapse (death as competing risk). Time to GVHD was defined as the period from HSCT to the onset of any‐grade disease. Survival probabilities were estimated using the Kaplan–Meier method. All variables presented in Table 1 were incorporated into the univariate analysis. Analyses were performed using SPSS version 16.0 (SPSS Inc., Chicago, IL). Final follow‐up occurred on April 30, 2025.

## Author Contributions

X.‐Y.Z. and X.‐J.H. designed the research. X.‐Y.Z., Z.‐L.X., and M.L. analyzed the data and wrote the manuscript. X.‐X.Y. and Y.‐Y.D. collected samples and performed flow cytometry. T.‐T.H., H.‐X.F., Y.‐Y.Z., X.‐D.M., Y.‐Q.S., L.‐P.X., X.‐H.Z., and Y.W. have provided the patient data. All authors have read and approved the final manuscript.

## Funding

This work was supported by Noncommunicable Chronic Diseases‐National Science and Technology Major Project (No. 2023ZD0501200), Beijing Outstanding Young Scientists Project (JWZQ20240101001).

## Ethics Statement

This study was approved by the Ethics Committee of Peking University People's Hospital (2016PHB006‐01), and the trial is registered at the US National Institutes of Health (NCT00539695).

## Conflicts of Interest

The authors declare no conflicts of interest.

## Data Availability

The data that support the findings of this study are available upon reasonable request from the corresponding author.

## References

[1] O. Penack , M. Marchetti , M. Aljurf , et al., “Prophylaxis and Management of Graft‐Versus‐Host Disease After Stem‐Cell Transplantation for Haematological Malignancies: Updated Consensus Recommendations of the European Society for Blood and Marrow Transplantation,” Lancet Haematology 11, no. 2 (2024): e147–e159. 38184001 10.1016/S2352-3026(23)00342-3

[2] S. Naik , Y. Li , A. C. Talleur , et al., “Memory T‐Cell Enriched Haploidentical Transplantation With NK Cell Addback Results in Promising Long‐term Outcomes: A Phase II Trial,” Journal of Hematology & Oncology 17, no. 1 (2024): 50. 38937803 10.1186/s13045-024-01567-0 PMC11212178

[3] V. D. Kheav , M. Busson , C. Scieux , et al., “Favorable Impact of Natural Killer Cell Reconstitution on Chronic Graft‐Versus‐Host Disease and Cytomegalovirus Reactivation After Allogeneic Hematopoietic Stem Cell Transplantation,” Haematologica 99, no. 12 (2014): 1860–1867. 25085354 10.3324/haematol.2014.108407 PMC4258747

[4] L. Ruggeri , M. Capanni , E. Urbani , et al., “Effectiveness of Donor Natural Killer Cell Alloreactivity in Mismatched Hematopoietic Transplants,” Science 295, no. 5562 (2002): 2097–2100. 11896281 10.1126/science.1068440

[5] A. Zafarani , M. Taghavi‐Farahabadi , M. H. Razizadeh , M. R. Amirzargar , M. Mansouri , and M. Mahmoudi , “The Role of NK Cells and Their Exosomes in Graft Versus Host Disease and Graft Versus Leukemia,” Stem Cell Reviews and Reports 19, no. 1 (2023): 26–45. 35994137 10.1007/s12015-022-10449-2

[6] M. Edinger , P. Hoffmann , J. Ermann , et al., “CD4+CD25+ Regulatory T Cells Preserve Graft‐Versus‐Tumor Activity While Inhibiting Graft‐Versus‐Host Disease After Bone Marrow Transplantation,” Nature Medicine 9, no. 9 (2003): 1144–1150. 10.1038/nm915 12925844

[7] S. Ito , C. M. Bollard , M. Carlsten , et al., “Ultra‐Low Dose Interleukin‐2 Promotes Immune‐Modulating Function of Regulatory T Cells and Natural Killer Cells in Healthy Volunteers,” Molecular Therapy: The Journal of the American Society of Gene Therapy 22, no. 7 (2014): 1388–1395. 24686272 10.1038/mt.2014.50 PMC4089007

[8] O. Boyman and J. Sprent , “The Role of Interleukin‐2 During Homeostasis and Activation of the Immune System,” Nature Reviews Immunology 12, no. 3 (2012): 180–190. 10.1038/nri3156 22343569

[9] A. I. Palamarchuk , E. I. Kovalenko , and M. A. Streltsova , “The hTERT and iCasp9 Transgenes Affect EOMES and T‐BET Levels in NK Cells and the Introduction of both Genes Improves NK Cell Proliferation in Response to IL2 and IL15 Stimulation,” Biomedicines 12, no. 3 (2024): 650. 10.3390/biomedicines12030650 PMC10968005 38540262

[10] V. Niederlova , O. Tsyklauri , M. Kovar , and O. Stepanek , “IL‐2‐driven CD8(+) T Cell Phenotypes: Implications for Immunotherapy,” Trends in Immunology 44, no. 11 (2023): 890–901. 37827864 10.1016/j.it.2023.09.003 PMC7615502

[11] B. C. Betts , J. Pidala , J. Kim , et al., “IL‐2 Promotes Early Treg Reconstitution After Allogeneic Hematopoietic Cell Transplantation,” Haematologica 102, no. 5 (2017): 948–957. 28104702 10.3324/haematol.2016.153072 PMC5477614

[12] A. A. Kennedy‐Nasser , S. Ku , P. Castillo‐Caro , et al., “Ultra Low‐Dose IL‐2 for GVHD Prophylaxis After Allogeneic Hematopoietic Stem Cell Transplantation Mediates Expansion of Regulatory T Cells Without Diminishing Antiviral and Antileukemic Activity,” Clinical Cancer Research 20, no. 8 (2014): 2215–2225. 24573552 10.1158/1078-0432.CCR-13-3205 PMC3989436

[13] X. Y. Zhao , X. S. Zhao , Y. T. Wang , et al., “Prophylactic Use of Low‐Dose Interleukin‐2 and the Clinical Outcomes of Hematopoietic Stem Cell Transplantation: A Randomized Study,” Oncoimmunology 5, no. 12 (2016): e1250992. 28123892 10.1080/2162402X.2016.1250992 PMC5215224

[14] D. Wolf , “GVHD Prophylaxis: Use an Ortho IL‐2/IL‐2Rbeta Treg System,” Blood 141, no. 11 (2023): 1246–1247. 36929438 10.1182/blood.2023019711

[15] X. D. Ma , Z. L. Xu , and X. J. Huang , “Immune Reconstitution After Haploidentical Hematopoietic Stem Cell Transplantation With Different Non‐T‐Cell Depletion Protocols,” MedComm 6, no. 6 (2025): e70206. 40391196 10.1002/mco2.70206 PMC12086379

[16] J. Koreth , K. Matsuoka , H. T. Kim , et al., “Interleukin‐2 and Regulatory T Cells in Graft‐Versus‐Host Disease,” New England Journal of Medicine 365, no. 22 (2011): 2055–2066. 22129252 10.1056/NEJMoa1108188 PMC3727432

[17] K. Matsuoka , H. T. Kim , S. McDonough , et al., “Altered Regulatory T Cell Homeostasis in Patients With CD4+ Lymphopenia Following Allogeneic Hematopoietic Stem Cell Transplantation,” The Journal of Clinical Investigation 120, no. 5 (2010): 1479–1493. 20389017 10.1172/JCI41072 PMC2860902

[18] J. Koreth , H. T. Kim , K. T. Jones , et al., “Efficacy, Durability, and Response Predictors of Low‐Dose Interleukin‐2 Therapy for Chronic Graft‐Versus‐Host Disease,” Blood 128 (2016): 130–137. 27073224 10.1182/blood-2016-02-702852 PMC4937358

[19] J. S. Whangbo , S. Nikiforow , H. T. Kim , et al., “A Phase 1 Study of Donor Regulatory T‐cell Infusion plus Low‐dose Interleukin‐2 for Steroid‐refractory Chronic Graft‐vs‐host Disease,” Blood Advances 6, no. 21 (2022): 5786–5796. 35475885 10.1182/bloodadvances.2021006625 PMC9647832

[20] T. Meyer , K. Maas‐Bauer , R. Wasch , et al., “Immunological Reconstitution and Infections After alloHCT—A Comparison Between Post‐transplantation Cyclophosphamide, ATLG and Non‐ATLG Based GvHD Prophylaxis,” Bone Marrow Transplantation 60, no. 3 (2025): 286–296. 39562716 10.1038/s41409-024-02474-1 PMC11893447

[21] B. Bielekova , M. Catalfamo , S. Reichert‐Scrivner , et al., “Regulatory CD56(Bright) Natural Killer Cells Mediate Immunomodulatory Effects of IL‐2Ralpha‐Targeted Therapy (Daclizumab) in Multiple Sclerosis,” Proceedings of the National Academy of Sciences of the United States of America 103, no. 15 (2006): 5941–5946. 16585503 10.1073/pnas.0601335103 PMC1458677

[22] V. D. Kheav , M. Busson , C. Scieux , et al., “Favorable Impact of Natural Killer Cell Reconstitution on Chronic Graft‐Versus‐Host Disease and Cytomegalovirus Reactivation After Allogeneic Hematopoietic Stem Cell Transplantation,” Haematologica 99, no. 12 (2014): 1860–1867. 10.3324/haematol.2014.108407 PMC4258747 25085354

[23] T. A. Fehniger , M. A. Cooper , G. J. Nuovo , et al., “CD56bright Natural Killer Cells Are Present in Human Lymph Nodes and Are Activated by T Cell‐Derived IL‐2: A Potential New Link Between Adaptive and Innate Immunity,” Blood 101, no. 8 (2003): 3052–3057. 12480696 10.1182/blood-2002-09-2876

[24] R. S. Goldszmid , P. Caspar , A. Rivollier , et al., “NK Cell‐derived Interferon‐Gamma Orchestrates Cellular Dynamics and the Differentiation of Monocytes Into Dendritic Cells at the Site of Infection,” Immunity 36, no. 6 (2012): 1047–1059. 22749354 10.1016/j.immuni.2012.03.026 PMC3412151

[25] Y. J. Chang , X. Y. Zhao , and X. J. Huang , “Effects of the NK Cell Recovery on Outcomes of Unmanipulated Haploidentical Blood and Marrow Transplantation for Patients With Hematologic Malignancies,” Biology of Blood and Marrow Transplantation 14, no. 3 (2008): 323–334. 18275899 10.1016/j.bbmt.2007.12.497

[26] K. Umino , K. Morita , T. Ikeda , et al., “Antibody‐Mediated Pathogenesis of Chronic GVHD Through DBY/HLA Class II Complexes and Induction of a GVL Effect,” Blood 142, no. 11 (2023): 1008–1021. 37363859 10.1182/blood.2023019799

[27] M. H. Sofi , L. Tian , S. Schutt , et al., “Ceramide Synthase 6 Impacts T‐Cell Allogeneic Response and Graft‐Versus‐Host Disease Through Regulating N‐RAS/ERK Pathway,” Leukemia 36, no. 7 (2022): 1907–1915. 35513703 10.1038/s41375-022-01581-6 PMC9256768

[28] W. H. Hallett , E. Ames , M. Alvarez , et al., “Combination Therapy Using IL‐2 and Anti‐CD25 Results in Augmented Natural Killer Cell‐mediated Antitumor Responses,” Biology of Blood and Marrow Transplantation: Journal of the American Society for Blood and Marrow Transplantation 14, no. 10 (2008): 1088–1099. 18804038 10.1016/j.bbmt.2008.08.001 PMC2735407

[29] V. Bachanova , S. Cooley , T. E. Defor , et al., “Clearance of Acute Myeloid Leukemia by Haploidentical Natural Killer Cells Is Improved Using IL‐2 Diphtheria Toxin Fusion Protein,” Blood 123, no. 25 (2014): 3855–3863. 24719405 10.1182/blood-2013-10-532531 PMC4064329

[30] M. R. Verneris , Natural Killer Cells and Regulatory T Cells: How to Manipulate a Graft for Optimal GVL (ASH Education Program Book, 2013). 10.1182/asheducation-2013.1.335 PMC4020013 24319201

[31] L. Q. Cao , W. X. Huo , X. H. Zhang , et al., “Peripheral Blood Stem Cell Transplantation From Haploidentical Related Donor Could Achieve Satisfactory Clinical Outcomes for Intermediate‐ or High‐Risk Adult Acute Myeloid Leukemia Patients,” Bone Marrow Transplantation 59, no. 2 (2024): 203–210. 37968447 10.1038/s41409-023-02117-x

[32] Q. Shang , L. Bai , Y. Cheng , et al., “Outcomes and Prognosis of Haploidentical Haematopoietic Stem Cell Transplantation in Children With FLT3‐ITD Mutated Acute Myeloid Leukaemia,” Bone Marrow Transplantation 59, no. 6 (2024): 824–831. 38443705 10.1038/s41409-024-02214-5

[33] Z. L. Xu , T. T. Han , X. L. Zhu , et al., “Randomized Trial of Anti‐Thymocyte Globulin Plus Lowdose Post‐transplant Cyclophosphamide to Prevent Graft‐Versus‐Host Disease in Haploidentical Transplantation,” Haematologica 110, no. 12 (2025): 2965–2973. 40534493 10.3324/haematol.2025.287504 PMC12666268

[34] Z. Xu , X. Mo , Y. Kong , et al., “Mini‐Dose Methotrexate Combined With Methylprednisolone as a First‐Line Treatment for Acute Graft‐Versus‐Host Disease: A Phase 2 Trial,” Journal of Translational Internal Medicine 11, no. 3 (2023): 255–264. 37662885 10.2478/jtim-2023-0111 PMC10474881

[35] Y. Wang , Q. F. Liu , D. P. Wu , et al., “Mini‐Dose Methotrexate Combined With Methylprednisolone for the Initial Treatment of Acute GVHD: A Multicentre, Randomized Trial,” BMC Medicine 22, no. 1 (2024): 176. 38664766 10.1186/s12916-024-03395-y PMC11044329

[36] X. Y. Pei , Q. Huang , L. J. Luo , et al., “Letermovir Prophylaxis for Cytomegalovirus Is Associated With Risk of Post‐Transplant Lymphoproliferative Disorders After Haploidentical Stem Cell Transplantation,” Haematologica 110, no. 4 (2025): 1005–1009. 39605206 10.3324/haematol.2024.286265 PMC11959248

[37] O. Penack , M. Marchetti , M. Aljurf , et al., “Prophylaxis and Management of Graft‐Versus‐Host Disease After Stem‐Cell Transplantation for Haematological Malignancies: Updated Consensus Recommendations of the European Society for Blood and Marrow Transplantation,” Lancet Haematology 11, no. 2 (2024): e147–e159. 10.1016/S2352-3026(23)00342-3 38184001

[38] M. H. Jagasia , H. T. Greinix , M. Arora , et al., “National Institutes of Health Consensus Development Project on Criteria for Clinical Trials in Chronic Graft‐Versus‐Host Disease: I. The 2014 Diagnosis and Staging Working Group Report,” Biology of Blood and Marrow Transplantation 21, no. 3 (2015): 389–401. e381. 25529383 10.1016/j.bbmt.2014.12.001 PMC4329079

